# Refinement of Triple-Negative Breast Cancer Molecular Subtypes: Implications for Neoadjuvant Chemotherapy Selection

**DOI:** 10.1371/journal.pone.0157368

**Published:** 2016-06-16

**Authors:** Brian D. Lehmann, Bojana Jovanović, Xi Chen, Monica V. Estrada, Kimberly N. Johnson, Yu Shyr, Harold L. Moses, Melinda E. Sanders, Jennifer A. Pietenpol

**Affiliations:** 1 Department of Biochemistry, Vanderbilt University, Nashville, Tennessee, United States of America; 2 Department of Medical Oncology, Dana-Farber Cancer Institute, Harvard University, Boston, MA, United States of America; 3 Division of Biostatistics, Department of Public Health Sciences, University of Miami Miller School of Medicine, Miami, FL, United States of America; 4 Sylvester Comprehensive Cancer Center, University of Miami Miller School of Medicine, Miami, FL, United States of America; 5 Department of Medicine, Breast Cancer Research Program, Vanderbilt University, Nashville, Tennessee, United States of America; 6 Center for Quantitative Sciences, Division of Cancer Biostatistics, Department of Biostatistics, Vanderbilt University, Nashville, Tennessee, United States of America; 7 Department of Cancer Biology, Vanderbilt University, Nashville, Tennessee, United States of America; 8 Department of Pathology, Microbiology and Immunology, Vanderbilt University, Nashville, Tennessee, United States of America; University of Torino, ITALY

## Abstract

Triple-negative breast cancer (TNBC) is a heterogeneous disease that can be classified into distinct molecular subtypes by gene expression profiling. Considered a difficult-to-treat cancer, a fraction of TNBC patients benefit significantly from neoadjuvant chemotherapy and have far better overall survival. Outside of BRCA1/2 mutation status, biomarkers do not exist to identify patients most likely to respond to current chemotherapy; and, to date, no FDA-approved targeted therapies are available for TNBC patients. Previously, we developed an approach to identify six molecular subtypes TNBC (TNBCtype), with each subtype displaying unique ontologies and differential response to standard-of-care chemotherapy. Given the complexity of the varying histological landscape of tumor specimens, we used histopathological quantification and laser-capture microdissection to determine that transcripts in the previously described immunomodulatory (IM) and mesenchymal stem-like (MSL) subtypes were contributed from infiltrating lymphocytes and tumor-associated stromal cells, respectively. Therefore, we refined TNBC molecular subtypes from six (TNBCtype) into four (TNBCtype-4) tumor-specific subtypes (BL1, BL2, M and LAR) and demonstrate differences in diagnosis age, grade, local and distant disease progression and histopathology. Using five publicly available, neoadjuvant chemotherapy breast cancer gene expression datasets, we retrospectively evaluated chemotherapy response of over 300 TNBC patients from pretreatment biopsies subtyped using either the intrinsic (PAM50) or TNBCtype approaches. Combined analysis of TNBC patients demonstrated that TNBC subtypes significantly differ in response to similar neoadjuvant chemotherapy with 41% of BL1 patients achieving a pathological complete response compared to 18% for BL2 and 29% for LAR with 95% confidence intervals (CIs; [33, 51], [9, 28], [17, 41], respectively). Collectively, we provide pre-clinical data that could inform clinical trials designed to test the hypothesis that improved outcomes can be achieved for TNBC patients, if selection and combination of existing chemotherapies is directed by knowledge of molecular TNBC subtypes.

## Introduction

Triple-negative breast cancer (TNBC) is a heterogeneous collection of breast cancers lacking expression of estrogen receptor (ER), progesterone receptor (PR), and human epidermal growth factor receptor 2 (HER2) amplification. Patients with TNBC have a higher risk of both local and distant recurrence and metastases are more likely to occur in the brain and lungs rather than bone compared to other breast cancers[1–4]. The majority of TNBC patients who progress to metastatic disease do so within the first three years after diagnosis, however, those patients who have not recurred during this time have similar survival rates as patients with ER-positive breast cancers [5–7]. Unlike ER-positive and HER2-amplified breast cancers, there is a lack of recurrent oncogenic driver alterations in TNBC [8–10]. This molecular heterogeneity has to date resulted in the absence of FDA-approved targeted therapies for TNBC

Chemotherapy is the main treatment option for patients with TNBC in the neoadjuvant, adjuvant or metastatic settings. Despite the rather aggressive clinical behavior of TNBC, approximately 30–40% of patients achieve a pathological complete response (pCR) with no histological evidence of disease at the time of surgery after neoadjuvant chemotherapy and those patients have much higher rates of survival[11,12]. However, patients that show evidence of residual disease after neoadjuvant chemotherapy are six times more likely to have recurrence and twelve times more likely to die of metastatic disease[1,12,13].

The differences in clinical response and survival after neoadjuvant chemotherapy suggest that a subset of TNBC may be inherently insensitive to cytotoxic chemotherapy. We and others have recently demonstrated that TNBCs are transcriptionally heterogeneous and can be grouped into subtypes with vastly differing biologies and responses to chemotherapy and targeted therapies [5,14–17]. Previously, we identified six molecular TNBC subtypes (TNBCtype)[14–16,18], each displaying unique ontologies and differential response to standard-of-care chemotherapy. The TNBC subtypes include two basal-like (BL1 and BL2), an immunomodulatory (IM), a mesenchymal (M), a mesenchymal stem-like (MSL), and a luminal androgen receptor (LAR) type[8,15,19]. The BL1 subtype is characterized by elevated cell cycle and DNA damage response gene expression, while the BL2 subtype is enriched in growth factor signaling and myoepithelial markers. The IM subtype is composed of genes encoding immune antigens and cytokine and core immune signal transduction pathways and likely represents gene expression from both the tumor cells and infiltrating lymphocytes. Both M and MSL subtypes share elevated expression of genes involved in epithelial-mesenchymal-transition and growth factor pathways, but only the MSL subtype has decreased expression of genes involved in proliferation. The LAR subtype is characterized by luminal gene expression and is driven by the androgen receptor (AR). In addition, we identified TNBC cell lines representative of each subtype and demonstrated differential sensitivity to alkylating agent cisplatin, with BL1 cell lines displaying the greatest sensitivity[15,19,20].

In a prior retrospective analysis of patient pretreatment biopsies, TNBCtype molecular subtypes were predictive of response to neoadjuvant anthracycline and cyclophosphamide followed by taxane (ACT), with BL1 subtype tumors exhibitng the highest pCR (52%) and BL2 and LAR subtypes the lowest (0 and 10%, respectively). These results suggest that certain TNBC subtypes are intrinsically sensitive or insensitive to neoadjuvant chemotherapy.

TNBCtype molecular subtypes were identified from surgical tumor specimens containing significant stromal and immune components including normal cells. To determine if normal cells contribute to TNBC subtypes, we performed histological evaluation, laser-capture microdissection, RNA isolation and gene expression analysis on a panel of TNBC tumors. We provide significant evidence that the IM and MSL TNBC subtypes represent tumors with substantial infiltrating lymphocytes and tumor-associated mesenchymal cells, respectively, and led us to refine our original TNBCtype (BL1, BL2, IM, M, MSL and LAR) to TNBCtype-4 (BL1, BL2, M and LAR).

Using the refined TNBCtype-4 on TNBC tumors genomically analyzed as part of The Cancer Genome Atlas (TCGA), we evaluated survival, age at diagnosis, grade, lymph node positivity, histopathological subtype enrichment and metastatic site preferences relative to TNBC subtype. In a retrospective analysis of gene expression datasets from five clinical trials we determined the predictive value of TNBCtype-4 subtypes in response to neoadjuvant chemotherapy.

## Materials and Methods

### TCGA breast cancer datasets and analysis

RNA-seq gene expression data for TCGA breast cancer (BRCA) study were obtained from the Broad GDAC Firehose (http://gdac.broadinstitute.org/). Gene level 3 RSEM mRNA expression (stddata_2015_02_04 run) was downloaded and used for bimodal identification of TNBC samples, PAM50 and TNBCtype subtyping. Mutation annotation files for BRCA were downloaded from GDAC Firehose (stddata_2015_02_04 run) and the total number of missense variants per sample determined. H&E stained sections corresponding to the biopsy or primary tumor used for gene expression were obtained from the cancer digital slide archive (http://cancer.digitalslidearchive.net/) for histological evaluation of lymphocytes. Level 1 clinical annotation was downloaded from GDAC Firehose (stddata_ 2015_02_04 run).

### TNBC587 microarray dataset

The TNBC587 dataset represents gene expression from 587 TNBC tumors extracted from 21 publically available Affymetrix microarray datasets and renormalized with each other as previously described[15,21]. The TNBC samples were identified by bimodal filtering of mRNA expression for ESR1, PGR and ERBB2 from each of the following datasets: GSE4394, GSE7904, GSE2109, GSE7390, GSE2990, GSE1456, GSE22513, GSE28796, GSE11121, GSE2603, GSE5364, GSE1561, GSE5327, GSE5847, GSE12276, GSE16446, GSE18864, GSE19615, GSE20194, MDA1333 and ETABM158.

### Patients, samples and clinical data

Retrospective analysis was performed on previously published, clinically annotated microarray datasets in the public domain (GSE25066[8], GSE41998[14], GSE22358[11], GSE22226[1] and GSE32646[5]) containing gene expression data from tumors of patients that received neoadjuvant chemotherapy (see Table 1 for details). Gene expression data were analyzed from pretreatment tumor samples. Pathological complete response (pCR) was defined as the absence of residual invasive adenocarcinoma in the breast and axillary lymph nodes upon histologic evaluation after neoadjuvant chemotherapy. All patients provided written informed consent and studies were approved by the Institutional Review Board or Independent Ethics Committee at all participating sites.

**Table 1:** Neoadjuvant chemotherapy dataset used for analyses.

Dataset (Trial)	Total patient # (with response)	TNBC patient # (with response)	TNBC (%)	Neoadjuvant Chemotherapy	Reference
GSE25066 (MDACC)	508 (488)	182 (176)	35.8	AT	Hatzis et al.
GSE22358 (XeNA)	154 (122)	62 (46)	40.3	Capecitabine + D	Glück et al.
GSE22226 (I-SPY-1)	149 (144)	34 (34)	22.8	AT	Essermann et al.
GSE32646 (Osaka)	115 (115)	31(31)	27.0	T + 5-FU/E/C	Miyake et al.
GSE41998 (BMS)	279 (273)	144 (130)	51.6	AC + 1:1 IXA or T	Horak et al.

5-FU, 5-fluorouracil; A, anthracycline; C, cyclophosphamide; D, docetaxel; E, epirubicin; IXA, ixabepilone; and T, taxane.

The Hatzis et al. dataset (GSE25066) generated by MD Anderson Cancer Center (MDACC)[8] consisted of 508 breast cancer gene expression profiles from HER2-negative breast cancer patients enrolled in a neoadjuvant chemotherapy trials that received an anthracycline-based and taxane regimen, either in combination or sequentially.

The Glück et al. dataset (GSE22358) contained gene expression profiles of tumors from 154 women with HER2-neu negative breast cancer that received chemotherapy alone consisting of 3 weekly cycles of treatment with Xeloda (capecitabine, 825 mg/m^2^) with taxotere (docetaxel, 75 mg/m^2^). Patients with HER2-neu positive breast cancer received the same chemotherapy in combination with Herceptin (trastuzumab)[11].

The Essermann et al. dataset (GSE22226) was obtained from pretreatment breast cancer biopsies from 149 women treated with four cycles of anthracycline followed optional taxane as per physician’s discretion [1].

The Miyake et al. dataset (GSE32646) gene expression profiles from pretreatment biopsies obtained from 115 women treated with 12 weekly cycles of paclitaxel (80 mg/m^2^) followed by 5-FU (500 mg/m^2^), epirubicin (75 mg/m^2^) and cyclophosphamide (500 mg/m^2^) every three weeks for four cycles[5].

The Horak et al. dataset (GSE41998) was obtained from the tumors of women enrolled on a randomized multicenter, phase II trial (NCT00455533) with no prior treatment and histologically confirmed primary invasive breast carcinoma regardless of hormone receptor or HER2 expression status[14]. Patients received sequential neoadjuvant therapy with 4 cycles of adriamycin (60 mg/m^2^) and cyclophosphamide (600 mg/m^2^), followed by a 1:1 randomization to either ixabeplione (40mg/m^2^ every 3 weeks for 4 cycles) or paclitaxel (80mg/m^2^ weekly for 12 weeks). Patients were stratified by tumor size at baseline, ER status, investigator site and clinical response to AC. All patients underwent surgery 4 to 6 weeks after the last dose of ixabepilone (n = 148) or paclitaxel (n = 147) and specimens evaluated by pathological review at each site. We obtained the publically available gene expression profiles from 279 pretreatment samples (GSE41998). In addition gene expression profiling was performed on tumor and adjacent stroma from 10 TNBC specimens from Vanderbilt University. All research involving human participants have provided written consent and the study approved by the Institutional Review Board (IRB090026).

### Gene expression microarray normalization and processing

Raw microarray expression (CEL) files were downloaded from Gene Expression Omnibus. The Affymetrix U133 PLUS 2.0 CEL files from GSE25066 and GSE32646 and U133A CEL files from GSE41998 were processed and normalized using Frozen Robust Multiarray Analysis (fRMA) implemented in Bioconductor package *frma*, which renders the samples from different studies comparable by utilizing information from the large publicly available microarray databases[19]. The log2-transformed gene expression values were the basis for the analysis presented in this study.

For datasets generated using Agilent microarray platforms (GSE22226 and GSE22358), processed gene expression data was obtained from the series matrix file in which Lowess-normalized data were obtained from the log2 ratio of sample (channel 2-Cy5) to the Stratagene human universal reference sample (channel 1-Cy3).

### Identification of TNBC patients from gene expression data

To compare mRNA expression with IHC results and eliminate potential false negative and include false positives, we approximated the empirical distributions ESR1, PGR and ERRBB2 mRNA expression from each dataset individually using a two-component Gaussian mixture (R *optim package)*. The following probe sets were used for each of the datasets: GSE41998, GSE25066 and GSE32646; ESR1 (205225_at), PGR (PR208305_at) and ERBB2 (216836_s_at), GSE22226 (GPL4133); ESR1_18336, PGR_2744, and ERBB2_43498, GSE22226 (GPL4133); ESR1_26884, PGR_6923, and ERBB2_37893, GSE22358; ESR1_26884, PGR_15163, and ERBB2_38777. Given the estimated distributions, the posterior probability of negative expression status of ER, PR and HER2 were determined and samples negative for expression of each of these markers identified.

### TNBCtype and PAM50 subtype predictor

For each dataset, all samples were analyzed using the PAM50 predictor using the robust method (R *genfu package*)[20]. To identify TNBC molecular subtypes, only TNBC samples that were determined by mixed Gaussian distribution were subtyped as individual datasets using TNBCtype (http://cbc.mc.vanderbilt.edu/tnbc/) as previously described[18].

### Histopathological evaluation of tumor lymphocytes from TCGA

The contribution of mononuclear chronic inflammatory cells to the cellularity of the entire tissue section was assessed histopathologically using digitally scanned H&E slides and Aperio software (Buffalo Grove, IL). Because non-microdissected tissues were used to generate the TCGA profiles, we assumed that peritumoral and intratumoral mononuclear cells were equally as likely to contribute to the gene expression profiles of individual tumors. Semi-quantitative assessment of the proportion of mononuclear cells was performed. The mononuclear cell infiltrate as a percentage of all nuclei in a field was characterized as mild (0–10%) moderate (20–40%) or intense (>50%), using a modification of the proportion score described by the International TILs Working Group[22].

### Gene expression analysis of laser capture-microdissected tumor and adjacent stroma

Depending on the amount of available tissue, laser capture microdissection (LCM) was performed on 30 to 50 sections (5μm) of frozen breast cancer needle core biopsies using the Arcturus PixCell IIe microscope (Mountain View, CA). RNA from LCM-captured tumor and adjacent stromal cells was isolated using the RNAqueous-Micro kit (Ambion, Grand Island, NY). RNA was validated for quality and subsequent cDNA synthesis and amplification performed (on 10 ng of total RNA) by the Vanderbilt Technologies for Advanced Genomics (VANTAGE) core. The reactions were run through first strand and second strand synthesis, followed by two rounds of single primer isothermal amplification (NuGEN, San Carlos, CA) amplification. The amplified cDNA product was hybridized to the Affymetrix HuGene 1.0ST array. Raw Affymetrix Human Gene CEL files were normalized using the Robust MultiChip Averaging (RMA) algorithm implemented in the Bioconductor package *Affy*. The probes were annotated using Bioconductor package *hugene10sttranscriptcluster*.*db* and the normalized, log2-transformed gene expression data used for further analysis. Gene expression data are available under GEO (Gene Expression Omnibus) accession number GSE81838.

### Statistical analysis

Odds ratio for pCR were computed for each TNBC and PAM50 subtype versus all unselected TNBC patients. Forest plots were generated by R package *rmeta*. Kaplan-Meier and log-rank tests were used to estimate and compare survival curves of TNBCs patients stratified by PAM50 or TNBCtype. 95% confidence intervals determined by R package *binom*. Chi-square tests were performed for all comparisons involving two categorical variables from a single population. Fisher’s exact test was performed on categorical variable comparisons between two groups. Wilcoxon signed rank test was used for pairwise significance testing of continuous variables. Cox proportional hazard model performed using IM correlation as continuous variable and significance determined by likelihood ratio test. All correlations use Spearman’s method. All statistical analyses were performed in R version 3.1.2.

## Results

### Significant correlation between IM subtype and the level of tumor infiltrating lymphocytes (TILs)

**Fig 1:**
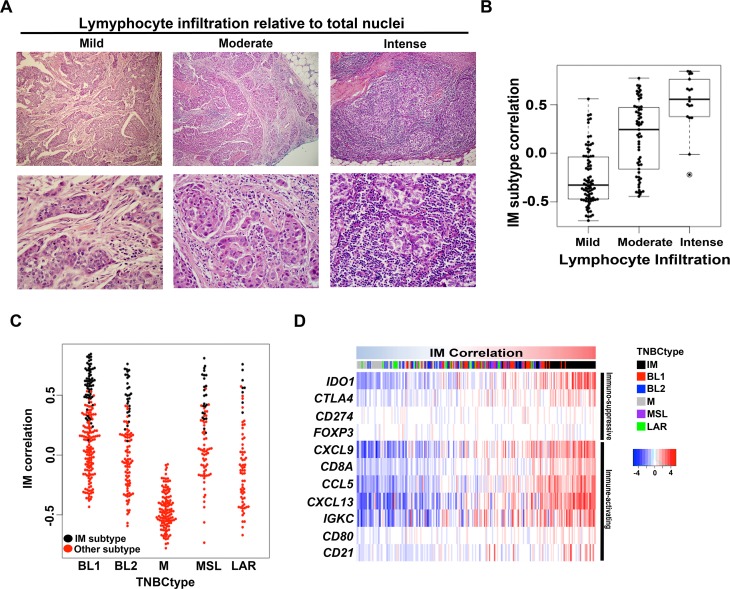
Analysis of lymphocyte infiltration and immune signaling gene expression in IM subtype TNBC. **(A)** Representative H&E images TNBC tumors that were scored for mild (0–10%) moderate (20–40%) or intense (>50%) levels of infiltrating lymphocytes relative to total nuclei. **(B)** Boxplot shows IM subtype gene expression correlation for each TCGA TNBC tumor binned into mild, moderate or intense levels by pathological evaluation of H&E slides. **(C)** Beeswarm plot shows the IM subtype correlation for 587 TNBC tumors according to TNBC subtypes (red). Tumors that were initially subtyped as IM, but have strong secondary correlations to other subtypes, are shown in black. **(D)** Heatmap shows expression of immuno-regulatory genes across 587 TNBC tumors ranked by increasing correlations to the IM TNBC centroid.

Gene expression profiles from human tumors are a composite, to varying degrees, of tumor and surrounding stromal and immune cells, including fibroblasts, adipocytes, endothelial, macrophages and lymphocytes. Recent studies have suggested prognostic value of TILs in TNBC[23,24]. Since the IM subtype is highly enriched for immune cell markers and signaling, we hypothesized that TIL levels in a tumor specimen would influence the IM subtype ‘call’ for a given TNBC. To test this hypothesis, we scored H&E sections from 180 TNBCs within The Cancer Genome Atlas (TCGA) for lymphocytes and analyzed the results relative to the TNBCtype call generated from the RNA-seq data (see methods). The study pathologists found that the percentage of total nuclei that represented by lymphocytes ranged from 0% to 70% with a median of 10% per tumor sample. The latter is considered mild lymphocytic presence (Fig 1A and S1 Table). Tumors classified as IM had the highest average percentage of lymphocytes with 38% followed by BL2 (23%), MSL (21%), LAR (17%), BL1 (15%) and M (9%) (S1 Table). Regardless of tumor subtype, the IM component for each tumor was highly correlated (Spearman, 0.67) with percentage lymphocytes. Analysis of the corresponding TNBCtype calls showed that the level of IM correlation was relative to the degree of lymphocytic presence when binned as mild (median = -0.32), moderate (median = 0.20) and intense (median = 0.56) lymphocytic presence (p<0.001) (Fig 1B).

To further examine the relative level of immune component in the other TNBC subtypes, we used a separate cohort of 587 TNBC tumors[15] and examined cases receiving a BL1, BL2, M, MSL and LAR primary ‘call’ for the presence of a secondary correlation to the IM subtype. BL1, BL2, MSL and LAR classified tumors all had representatives with high secondary correlations to the IM subtype (Fig 1C). In contrast, mesenchymal (M) classified tumors all have a very low IM correlation. In fact there is a negative correlation (Spearman, -0.95) between IM and M across all tumors (S1A Fig), suggesting opposite biological states with M-subtyped tumors having a microenvironment that is non-permissive to immune cell infiltration or immunosuppressed (S1B Fig). These data provide strong evidence that the infiltrating lymphocytes contribute significantly to the gene expression profiles for the IM subtype and that correlations to this signature should be considered as a descriptor of the immune state of the tumor rather than an independent subtype[25].

A previously published gene signature composed of T- and B-cell markers, chemokines, and immune checkpoint regulators (*CXCL9*, *CCL5*, *CD8A*, *CD80*, *CXCL13*, *IGKC*, *CD21*, *IDO1*, *PD-1*, *PD-L1*, *CTLA4*, *FOXP3*) was shown to be highly correlated with pathological evaluation of immune cell infiltrate[23]. To further demonstrate that the IM subtype represents tumors with high lymphocytic infiltrate, we examined the expression of the genes listed above in the gene expression data set of 587 TNBC[15]. When ordered by increasing correlation to the IM subtype, high immune gene expression was confined IM, BL1, BL2 and MSL tumors, with very low expression in M tumors (Fig 1D). IM subtype tumors had high levels of the immune checkpoint regulatory genes *CTLA4* CD274 (the gene encoding PD-L1) and *PDCD1* (the gene encoding PD-1) and may be amenable to agents targeting immune checkpoints given the recent success of anti-PD-1 and anti-PD-L1 therapy in TNBC [26].

### Significant correlation between MSL TNBC subtype and the level of stromal mesenchymal cell gene expression

**Fig 2:**
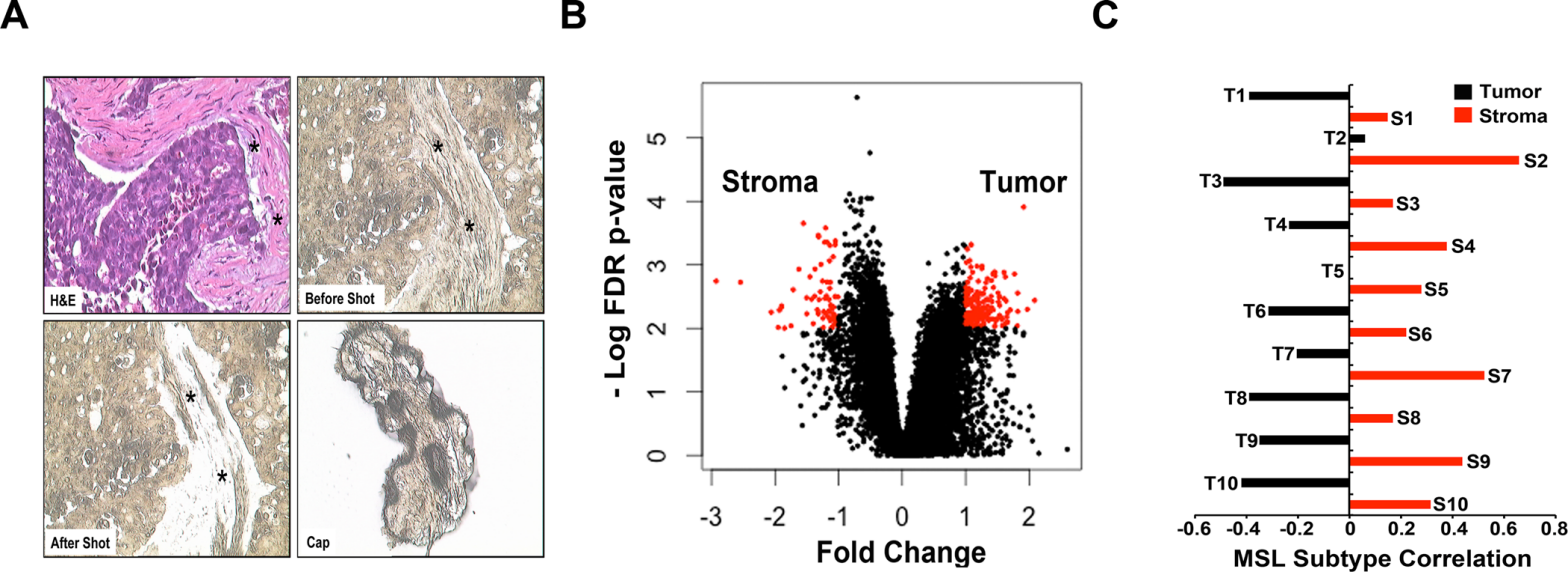
LCM followed by gene expression analysis of tumor epithelium and adjacent stroma identifies normal stromal cell gene expression in the MSL TNBC subtype. **(A)** Representative images of H&E (upper left), tissue before (upper right) and after (lower left) LCM and cells isolated (lower right) for gene expression analysis in adjacent tumor stroma (asterisk). **(B)** Scatter-plot shows differentially expressed genes (FC> 2, FDR< 0.01) between LCM-isolated tumor epithelium and stromal cells from 10 TNBC tumors. **(C)** Bar-plot shows the correlation of each tumor epithelial and stromal pairs to the MSL subtype.

We performed laser-capture microdissection (LCM) on 10 TNBC tumors followed by RNA isolation and gene expression analysis on malignant epithelial cell-enriched areas and the adjacent stromal cell-containing areas of the tumor sections (Fig 2A). Principal component analysis demonstrated that overall gene expression profiles were more similar within tumor samples and stromal samples than between matched tumor/stromal samples (S2 Fig). To determine if we efficiently captured tumor and stromal cells, we identified differentially expressed genes (fold change, FC> 2, false discovery rate, FDR< 0.01) between tumor and stromal samples and performed gene set enrichment analysis (S2 Table and Fig 2B). Among the most significantly enriched pathways in dissected, tumor epithelial cells were cell cycle, mitosis, DNA replication, cell cycle checkpoint and G1/S transition. In contrast, dissected stromal samples were highly enriched for expression of genes encoding extracellular matrix proteins, collagens, proteoglycans, glycoproteins and integrins (Table 2). Analysis of TNBC subtypes from both the matched tumor epithelium and stroma revealed that six of ten pairs had discordant subtype calls (Table 3). Of these, five changed to MSL when the stromal gene expression was analyzed, suggesting that the MSL subtype has features of surrounding cells or that these samples are comprised of stromal gene expression. Examination of the centroid correlations between each of the pairs revealed that the correlations remained stable for all subtypes, with exception of MSL (S2 Table). The MSL component is significantly higher for the adjacent stromal cells compared to the tumor epithelium for each of the pairs (Wilcoxon signed-rank p = 0.001953), indicating the MSL subtype is comprised of tumors with a high abundance of tumor-associated mesenchymal tissue (Fig 2C).

**Table 2:** Gene ontology analysis of differentially expressed genes between tumor epithelial and stromal cell.

**Gene ontology pathways enriched in tumor epithelium**			
**Gene Set Name**	**Description**	**# Genes in overlap**	**FDR q-value**
Reactome cell cycle	Genes involved in Cell Cycle	21	1.21E-14
Reactome cell cycle mitotic	Genes involved in Cell Cycle, Mitotic	19	1.28E-14
Reactome mitotic M G1	Genes involved in Mitotic M-M/G1 phases	13	4.38E-11
Reactome DNA replication	Genes involved in DNA Replication	13	1.35E-10
KEGG cell cycle	Cell cycle	10	1.44E-08
Reactome cell cycle checkpoints	Genes involved in Cell Cycle Checkpoints	9	2.17E-07
Reactome mitotic prometaphase	Genes involved in Mitotic Prometaphase	8	2.45E-07
Reactome mitotic G1/S phases	Genes involved in Mitotic G1-G1/S phases	9	3.95E-07
Reactome g1 s transition	Genes involved in G1/S Transition	8	1.44E-06
PID plk1 pathway	PLK1 signaling events	6	2.49E-06
**Gene ontology pathways enriched in tumor stoma**			
**Gene Set Name**	**Description**	**# Genes in overlap**	**FDR q-value**
NABA matrisome	Genes encoding ECM proteins	22	2.32E-16
NABA core matrisome	Core ECM glycoproteins and collagens	14	6.39E-15
NABA proteoglycans	Genes encoding proteoglycans	5	1.06E-06
Reactome collagen formation	Genes involved in Collagen formation	5	1.09E-05
PID integrin1 pathway	Beta1 integrin cell surface interactions	5	1.68E-05
PID avb3 integrin pathway	Integrins in angiogenesis	5	2.68E-05
Reactome extracellular matrix	Genes involved in ECM organization	5	4.85E-05
NABA collagens	Genes encoding collagen proteins	4	1.13E-04
PID syndecan 1 pathway	Syndecan-1-mediated signaling events	4	1.20E-04
KEGG melanoma	Melanoma	4	6.25E-04

**Table 3:** TNBC subtype correlations from matched LCM tumor epithelial.

Pair ID	Tissue	TNBCtype	Match
T1	tumor	BL1	FALSE
S1	stroma	MSL	
T2	tumor	BL1	FALSE
S2	stroma	MSL	
T3	tumor	BL1	FALSE
S3	stroma	MSL	
T4	tumor	BL1	FALSE
S4	stroma	MSL	
T5	tumor	BL2	TRUE
S5	stroma	BL2	
T6	tumor	M	FALSE
S6	stroma	BL2	
T7	tumor	M	TRUE
S7	stroma	M	
T8	tumor	MSL	TRUE
S8	stroma	MSL	
T9	tumor	M	FALSE
S9	stroma	MSL	
T10	tumor	LAR	TRUE
S10	stroma	LAR	

### Clinical, histological and genomic differences in refined TNBC subtypes

Having demonstrated that IM and MSL subtype calls are strongly weighted by stromal cell gene expression and subtype correlations are independent of one another, we refined TNBCtype from six to four subtypes (TNBCtype-4) by re-assigning IM and MSL subtypes to the second highest correlated centroid. Using PAM50, TNBCtype and the refined TNBCtype-4 subtyping algorithms we reanalyzed 587 TNBC tumors from publically available gene expression data[15] and 180 additional cases from TCGA[9] for clinical, histological and genomic differences. Given the similar distribution of subtypes for TNBCtype, TNBCtype-4 and PAM50, we merged TNBC patients from both datasets and analyzed clinical variables (S3 Table).

**Fig 3:**
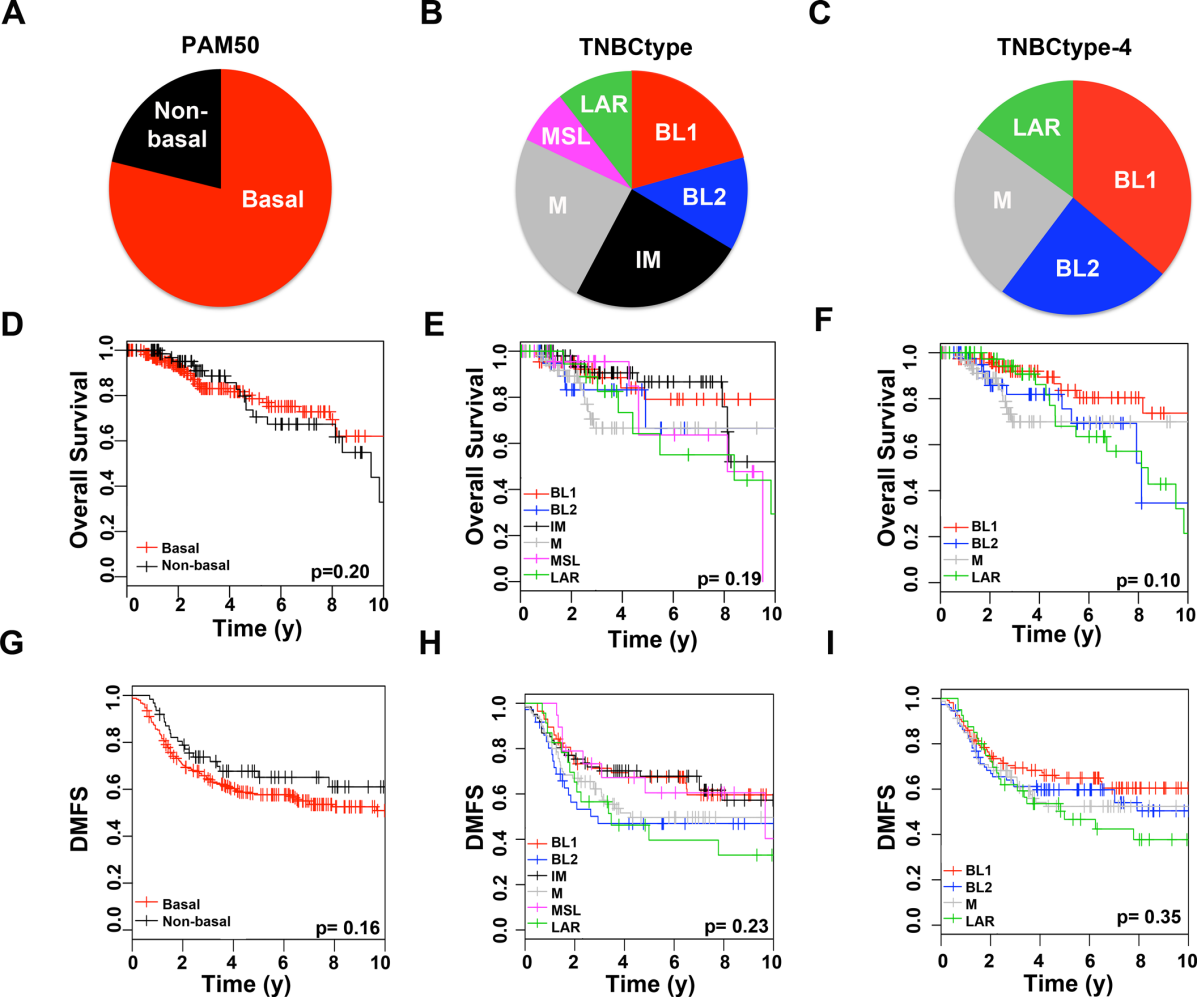
Molecular subtype distribution and survival analysis of TNBC samples stratified by PAM50, TNBCtype or refined TNBCtype-4. Piecharts show the distribution of 767 TNBC samples by **(A)** PAM50 **(B)** TNBCtype or **(C)** refined TNBCtype-4. Kaplan-Meier curves show overall survival for TNBC patients stratified by **(D)** PAM50 **(E)** TNBCtype or **(F)** refined TNBCtype-4 or relapse-free survival stratified by **(G)** PAM50 **(H)** TNBCtype or **(I)** refined TNBCtype-4. P-values shown were determined by logrank test. * indicates significant (p<0.05) pairwise survival differences between a subtype and all other subtypes combined not adjusted for multiple comparisons.

When analyzed using PAM50 subtyping, the majority of tumors were basal (n = 592, 77%), with lower fractions being of the HER2 (n = 91, 12%), LumA (n = 34, 4%), normal-like (n = 31, 4%) and LumB (n = 19, 3%) subtypes (S3 Table). Since there were relatively few samples in each PAM50 subtype outside of basal, we merged all non-basal subtypes (n = 175, 23%) (Fig 3A)[27].

Subtyping by the original TNBCtype resulted in a distribution of 18% BL1, 11% BL2, 21% IM, 21% M, 8% MSL, 9% LAR and 12% unclassified (Fig 3B). Classification by TNBCtype-4 resulted in a distribution of 35% BL1, 22% BL2, 25% M, 16% LAR and 2% unclassified Fig 3C). The distribution of PAM50 subtype “calls” among the TNBC subtypes showed that the majority of BL1, BL2, and M were basal-like, while LAR were enriched in HER2 and luminal subtypes (S4 Table).

Overall survival (OS) and relapse-free survival (RFS) for TNBC patients were analyzed by PAM50, TNBCtype and refined TNBCtype-4 to determine if molecular subtypes are associated with survival outcomes. There were no significant differences in overall survival for TNBC patients when stratified by PAM50 (p = 0.20), TNBCtype (p = 0.19) or TNBCtype-4 (p = 0.10) (Fig 3D–3F). However, overall survival for BL1 patients was significantly better than all other TNBCtype-4 subtypes combined (p = 0.037) (Fig 3F).

Relapse-free survival did not significantly differ in patients stratified by PAM50 (p = 0.16), TNBCtype (p = 0.23), and TNBCtype-4 (p = 0.35) (Fig 3G–3I). When stratified by PAM50, non-basal TNBC patients tended to have better RFS survival than basal TNBC, however at 10 years follow up, neither reached median survival (Fig 3G). Stratification of patients by TNBCtype resulted in three subtypes in which median RFS was reached before 5 years (BL2 1.58, LAR 3.47 and M 4.25), in contrast to BL1, IM and MSL (Fig 3H). BL1 patients displayed better relapse-free survival with near 60% survival even at 10 years when stratified by TNBCtype or TNBCtype-4 (Fig 3H and 3I).

The IM subtype displayed the best overall and relapse-free survival (Fig 3E and 3H). Since the IM subtype had a higher amount of lymphocytes and better survival, we examined whether correlation to the IM centroid resulted in better survival, regardless of TNBCtype subtype. COX proportional hazard modeling survival using correlation to the IM centroid as a continuous variable demonstrated significant increases in relapse-free survival (likelihood ratio p = 0.0494) and trend for increased overall survival (likelihood ratio p = 0.0742) (S3 Fig). Therefore the presence of lymphocytes as measured by the IM correlation has predictive value for better relapse-free survival for TNBC patients, regardless of TNBCtype subtype.

To determine if PAM50 or the refined TNBCtype-4 subtypes display clinical differences, we examined age of diagnosis, tumor size, grade and lymph node involvement among the subtypes from annotated microarray gene expression and TCGA RNA-seq data (Table 4). Non-basal TNBC tumors were diagnosed in older women relative to basal TNBC (58.5 vs. 52.6, p = 3.38E-6). The LAR subtype was diagnosed in women of older ages compared to all other TNBCtype subtypes (59.5 vs. 52.7, p = 3.08E-5). Tumor size did not significantly vary within PAM50 (p = 0.6201) or TNBC subtypes (p = 0.4265). However, tumor grade was significantly different in TNBC patients stratified by PAM50, with basal TNBC tumors more likely to be of higher grade than non-basal TNBC (p = 0.0004). TNBCtype-4 subtyping had significant (p = 0.0003) differences in grade, with BL1 tumors being of higher grade and LAR tumors lower grade.

**Table 4:** Clinical parameters of TNBC subtypes.

	**Age**					**p-value**
***TNBCtype***						p = 3.08E-05
BL1	50.4					
BL2	52.6					
M	52.4					
LAR	60.6					
***PAM50***						p = 1.97E-06
Basal	51.4					
Non-basal	59.3					
	**Tumor Size (mm)**					**p-value**
***TNBCtype***						p = 0.4265
BL1	25.2					
BL2	27.4					
M	23.9					
LAR	25.3					
***PAM50***						p = 0.6201
Basal	25.2					
Non-basal	26.5					
	**Grade**					
	**N =**	**1**	**2**	**3**		**p-value**
***TNBCtype***						p = 0.0003038
BL1	112	0 (0.0)	14 (12.5)	98 (87.5)		
BL2	70	2 (2.9)	12 (17.1)	56 (80.0)		
M	84	1 (1.2)	23 (27.4)	60 (71.4)		
LAR	50	5 (10.0)	14 (28.0)	31 (62.0)		
***PAM50***						p = 0.0003668
Basal	247	3 (1.2)	41 (16.6)	203 (82.1)		
Non-basal	75	5 (6.6	24 (32.0)	48 (64.0)		
	**Stage**					
	**N =**	**1**	**2**	**3**	**4**	**p-value**
***TNBCtype***						p = 0.0082
BL1	58	7 (12.1)	47 (81.0)	4 (6.9)	0 (0.0)	
BL2	33	6 (18.2)	17 (51.5)	10 (30.3)	0 (0.0)	
M	44	9 (20.5)	30 (68.2)	3 (6.8)	2 (4.5)	
LAR	36	8 (22.2)	20 (55.6)	8 (22.2)	0 (0.0)	
***PAM50***						p = 0.0004
Basal	129	19 (14.7)	90 (69.8)	18 (13.9)	2 (1.6)	
Non-basal	46	13 (28.3)	25 (54.3)	8 (17.4)	0 (0.0)	
	**Lymph Node Positive**					
	**No**	**Yes**	**Incidence**			**p-value**
***TNBCtype***						p = 0.02778
BL1	73	35	0.32			
BL2	53	26	0.33			
M	66	18	0.21			
LAR	25	22	0.47			
***PAM50***						p = 0.7347
Basal	175	78	0.31			
Non-basal	49	25	0.34			

In contrast to lower histological grade, non-basal TNBC presented with significantly more advanced clinical disease and higher stage than basal TNBC (p = 0.0004; Table 4). Stratification by TNBCtype-4 resulted in significant differences in disease stage (p = 0.0003). Despite being of histologically higher grade, BL1 (6% stage 3) tumors were of lower clinical stage than BL2 (30% stage 3) and LAR (22% stage) tumors. Regional spread to lymph nodes occurred in 34% of TNBC and there was no significant difference between basal (29%) and non-basal (31%) TNBC (p = 0.1325). However, there was a significant enrichment of lymph node metastasis in LAR TNBC, with nearly half (47%) of these patients displaying regional spread (p = 0.0278; Table 4). Lymph node involvement was lower for the M TNBC subtype, as only 21% had lymph node disease.

Since there are clear differences in regional lymph node spread, we evaluated if these differences resulted in differential clinical progression. TNBCs have been shown to have high frequency of lung and brain metastases[28]. Using published datasets with metastasis-site annotations (GSE12276, GSE2034 and GSE2603), we identified 124 patients with site-specific metastasis data and examined the metastatic pattern in TNBC subtypes[29]. Overall in TNBC, the incidence of brain (11%), bone (19%) and lung (31%) metastasis were similar to a previous report of 10.9%, 16.6% and 18.5% to brain, bone and lung, respectively[28]. Stratification by TNBCs subtype did not show any statistical differences in brain (p = 0.1238) and lung (p = 0.0776) metastasis (S5 Table). However, the M subtype displayed a significantly higher frequency of lung metastasis (46%) compared to all other subtypes (25%) (p = 0.0388). Metastasis to the bone was significantly different among TNBC subtypes (p = 0.0398). For example, the incidence of bone metastasis was significantly higher for the LAR subtype (46%) as compared to all other subtypes 16% (p = 0.0456), consistent with the preference of hormonally-regulated cancers to metastasize to bone [30].

Since stratification by TNBCtype-4 resulted in subtypes with clinical differences, we examined if stratification by PAM50 or TNBCtype-4 subtypes enriched for atypical histology within TCGA cohort (S6 Table). Nearly all of the special histological subtypes are basal by PAM50, with exception of the lobular carcinomas that are luminal and the secretory and an invasive pleomorphic lobular carcinoma that are normal-like (S6 Table). While comprising the largest TNBCtype-4 subtype, BL1 tumors were largely ductal carcinomas without notable atypical histology. In contrast, infiltrating lobular carcinomas were nearly exclusive to the LAR subtype (4 of 5), suggesting a potential role for AR signaling in lobular breast cancer. Medullary carcinomas are characterized by infiltrating carcinomas with circumscribed pushing borders, dense peripheral lymphoid infiltrate and have favorable outcome. Medullary breast cancer histological types were present in BL1, BL2 and LAR and absent in the M subtype, consistent with the lack of lymphocytic infiltration in the M subtype. Metaplastic carcinomas display differentiation towards squamous epithelium with mesenchymal components and cells displaying spindle, chondroid, osseous or rhabdoid morphologies. All of the metaplastic carcinomas were either BL2 (n = 4) or M (n = 4), with one BL2 described as squamous. In, contrast, each of the metaplastic breast cancers are classified as basal by PAM50, even though they display striking differences in morphology.

### Analysis of over 300 TNBC patients treated with neoadjuvant chemotherapy identifies TNBC molecular subtypes with differing responses

**Fig 4:**
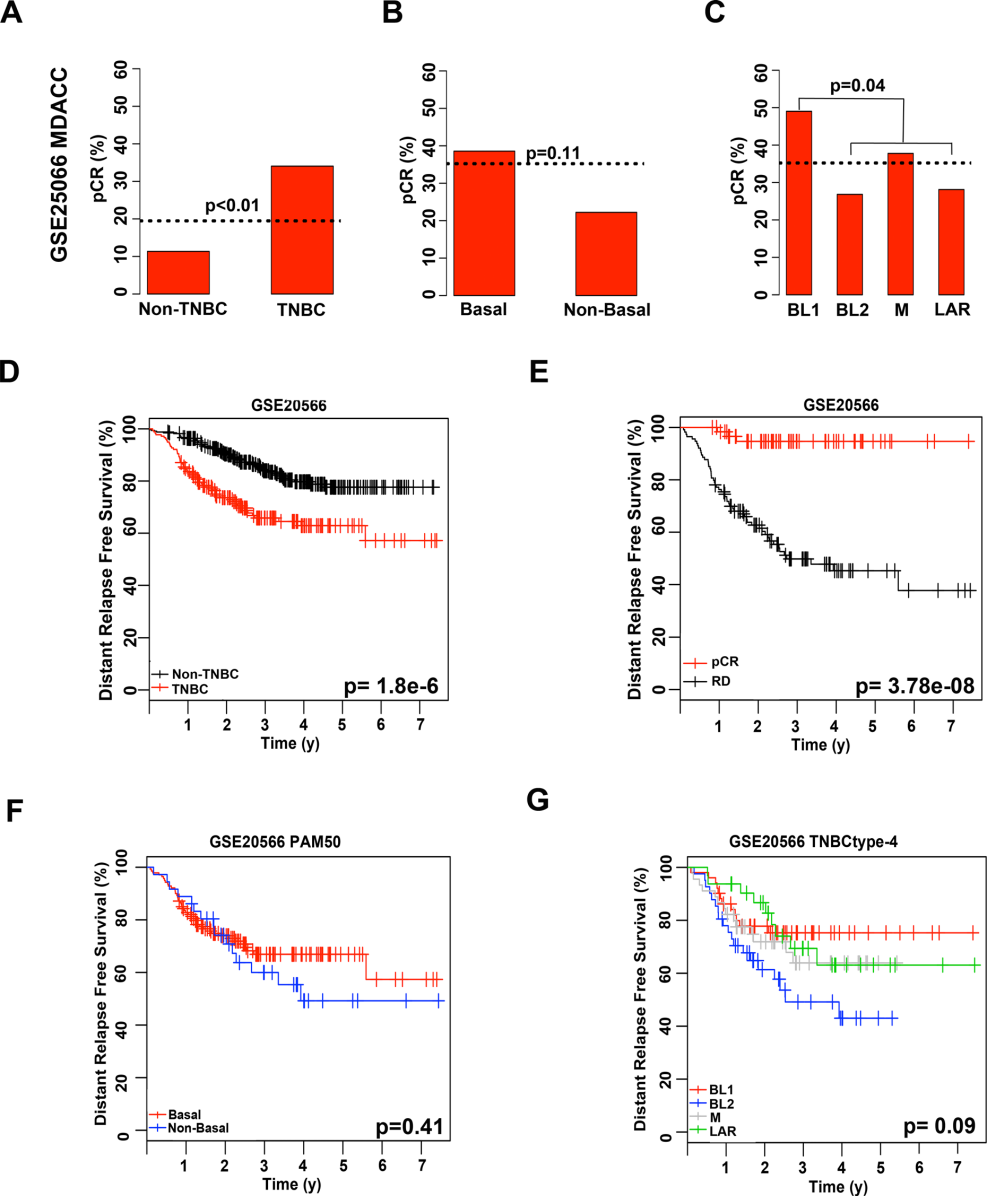
Chemotherapy response and distant relapse-free survival of TNBC treated with neoadjuvant anthracycline and taxane relative to PAM50 or refined TNBCtype-4 subtyping. Barplots show pCR rates achieved for patients stratified by **(A)** TNBC, **(B)** PAM50 or **(C)** TNBCtype-4. Dotted horizontal line indicates pCR for the individual cohort. Statistical significance determined by Fisher’s exact test. Kaplan-Meier plots display distant relapse-free survival from GSE25066 for **(D)** TNBC patients; **(E)** TNBC patients stratified by pCR or RD; **(F)** TNBC patients stratified by PAM50; and **(G)** TNBC patients stratified by refined TNBCtype-4.

Masuda and colleagues previously showed that TNBC patients significantly differ in response to neoadjuvant chemotherapy composed of anthracycline and taxane (A-T) based on the subtype of their tumor as determined by TNBCtype[13]. To determine if women with the refined TNBCtype-4 subtypes have differing outcomes to standard chemotherapy, we re-examined the MDACC cohort (GSE25066) used by Masuda et al.[13] with PAM50, and the refined TNBCtype-4 (S7 Table). To identify TNBC patients within each of the cohorts, we applied a mixed Gaussian distribution for each dataset using the mRNA expression for ER, PR and ERBB2 (S4 Fig). Using mixed Gaussian distribution along with annotated pathological calls, we were able to identify 182 TNBC patients, of which 176 had neoadjuvant chemotherapy response information. Consistent with previous reports, TNBC patients had higher pCR than non-TNBC patients (34% vs. 11%; p = 0.0001), in the GE25066 dataset (Fig 4A)[12]. TNBC tumors were subtyped by either PAM50 or TNBCtype-4 and chemotherapy response evaluated in this dataset. PAM50 subtyping of tumors in to basal and non-basal subtypes did not result in significant differences in pCR (p = 0.1135; Fig 4B). TNBCtype-4 subtyping did not result in significant differences in pCR for TNBC patients treated with neoadjuvant chemotherapy (p = 0.1074; Fig 4C), the pCR incidence for the subtypes displayed shows similar trends to previous studies, with BL1 displaying the greatest response and BL2 and LAR with lower pCR. However, compared to all other subtypes, BL1 patients had significantly higher pCR (49% vs. 31%; p = 0.0441).

Distant relapse-free survival was evaluated in the same cohort to determine if chemotherapy responses to neoadjuvant ACT resulted in differences in survival within PAM50 and TNBCtype-4 subtypes. Despite having better pCR to neoadjuvant chemotherapy (34% vs. 11%), TNBC patients had significantly worse DRFS survival compared to non-TNBC (p = 1.8e-6; Fig 4D). However, TNBC patients that responded to chemotherapy and achieved a pCR clearly had a far better DRFS compared to those patients that did not, with 95% of patients surviving seven years after treatment compared to a median survival of 2.7 y (p = 3.78e-8; Fig 4E). While there were no differences in DRFS between basal and non-basal PAM50 subtypes (p = 0.41), stratification by TNBCtype-4 trended towards significance (p = 0.09), with BL2 patients displaying the worst outcome with a median survival of 2.4 y compared to a median survival for unselected TNBC being greater than 7 y (Fig 4F and 4D). In contrast, the BL1 subtype displayed the highest pCR (49%) and also the best long-term DRFS with 72% of patients relapse-free at 7 y follow up (Fig 4G).

To determine if TNBC patients vary in their response to neoadjuvant chemotherapy by subtype, we evaluated data from four additional neoadjuvant chemotherapy datasets (GSE22358, GSE32464, GSE22226, GSE41998). Using a mixed Gaussian distribution, we were able to identify 46 (GSE22358), 34 (GSE22226), 31 (GSE32646) and 144 (GSE41998) TNBC patients from each dataset (Table 1). TNBC patients displayed higher pCR than non-TNBC patients across each dataset (GSE22358; 17% vs. 6%, GSE22226; 32% vs. 19%, and GSE32646; 36% vs. 19% and GSE41998; 36% vs. 15%, regardless of chemotherapy regimens (S5 Fig). TNBC patients were then subtyped by either TNBCtype-4 or PAM50 and response to chemotherapy evaluated in each dataset (S7 Table). There were no significant differences in pCR to neoadjuvant chemotherapy for either PAM50 or TNBCtype-4 subtypes in each data set alone (S5 Fig). However, there were similar distributions of response in datasets receiving similar chemotherapy regimens (GSE25066, GSE22226, GSE22358 and the paclitaxel arm of GSE41998).

**Fig 5:**
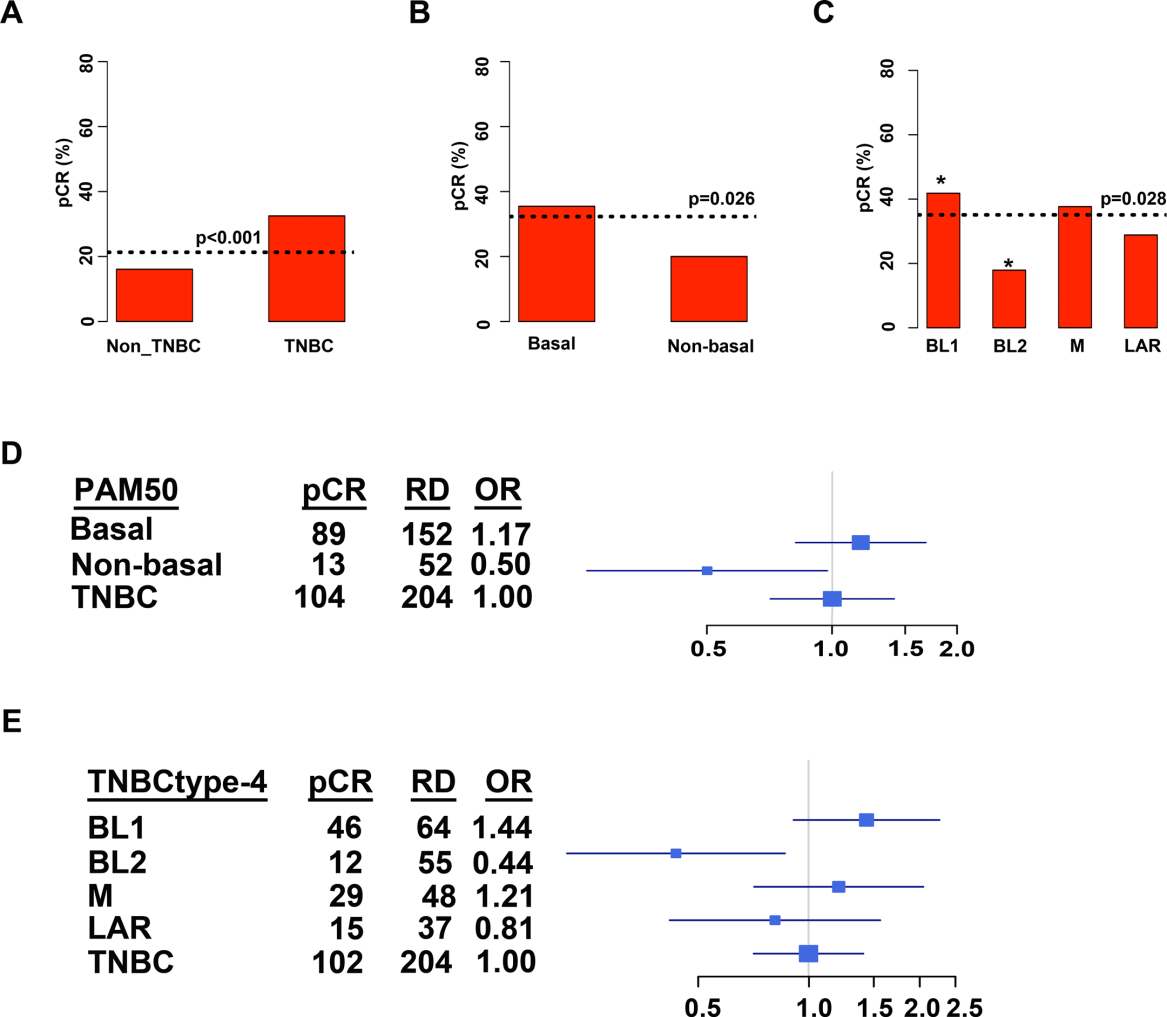
Combined retrospective analyses of 306 TNBC tumors treated with neoadjuvant chemotherapy stratified by molecular subtype. Barplots show pCR for **(A)** all breast cancer stratified by non-TNBC and TNBC or **(B)** TNBC patients stratified by PAM50 or **(C)** TNBCtype-4. Dotted horizontal lines indicate pCR for the stratified individual cohort. Table shows distribution of pCR, residual disease (RD) and odds ratio (OR) for a pCR in TNBC patients stratified by **(D)** PAM50 or **(E)** TNBCtype-4. Forrest plots display OR for pCR in subtypes relative to all TNBC.

Since none of the datasets alone reached statistical significance, we combined datasets (GSE25066, GSE22226, GSE22358 and the paclitaxel arm of GSE41998) that displayed similar responses to similar classes of neoadjuvant chemotherapy (S7 Table). In the combined cohort of 306 TNBC patients, the overall response to neoadjuvant chemotherapy was 21%, with TNBC tumors having a higher pCR than non-TNBC (33% vs. 16%, p = 0.0001; Fig 5A). Analysis of PAM50 subtypes resulted in majority of TNBC classified as basal (n = 264; 80%) compared to non-basal (n = 68; 20%), similar to previous reports[27]. Basal TNBC tumors had a greater response to neoadjuvant chemotherapy than non-basal TNBC (36% vs. 20%, p = 0.0175; Fig 5B). Subtyping with TNBCtype resulted in a distribution of 36% BL1, 22% BL2, 25% M and 17% among the TNBC subtypes (S6 Table). Stratification of pretreatment biopsies by TNBCtype showed significant (chi-square p = 0.0282) differences in chemotherapy response, with pCR for BL2 (18%) far below unselected TNBC (33%) or even PAM50 non-basal TNBC (20%) (Fig 5C). In contrast, tumors stratified into the BL1 subtype had a greater pCR (41%) compared to unselected TNBC (33%) or PAM50 basal TNBC (36%) (Fig 5C). Compared to all other subtypes, BL1 had a significantly greater rate of pCR (p = 0.0256), while the BL2 subtype had a significantly lower rate of pCR (p = 0.0185).

To determine how likely TNBC patients were to achieve a pCR, we computed the odds ratio for pCR for each subtype compared to all TNBC patients. In the combined dataset of 306 TNBC patients, unselected TNBC patients were 2.5 times more likely to achieve pCR than non-TNBC. Stratifying TNBC by PAM50 into basal (OR, 1.17) was slightly more likely to achieve pCR while non-basal TNBC (OR, 0.50) was less likely to achieve pCR than unselected TNBC (OR, 1.00) (Fig 5D). Stratification of tumors by TNBCtype resulted in BL1 (OR, 1.44) and M (OR, 1.21) tumors with greater odds and both BL2 (OR, 0.44) and LAR (OR, 0.81) tumor with lower odds of achieving a pCR compared to unselected TNBC (OR, 1.00) (Fig 5E). Stratification of TNBC patients by refined TNBCtype-4 could identify those patients most and least likely to respond to neoadjuvant chemotherapy. These findings support the need for further clinical testing of the predictive power of TNBC molecular subtyping.

## Discussion

TNBC is a molecularly heterogeneous disease that we had previously subtyped by gene expression into six different subtypes[15]. Herein we refine this prior classification into four subtypes based after taking into consideration the contribution of transcripts from normal stromal and immune cells in the tumor environment. The four TNBC subtypes display differing clinical characteristics with BL1 tumors displaying higher grade, lower stage and increased patient overall and relapse-free survival. TNBC subtypes displayed different patterns of progression with patients with LAR tumors having increased regional spread and preferential distant metastasis to bone, while M tumors preferentially metastasize to lung. Clinical differences were complimented by histological differences, with lobular carcinomas exclusive to the LAR subtype and metaplastic carcinomas either M or BL2. More importantly, TNBC subtypes differed in their response to standard neoadjuvant chemotherapy, with BL1 subtype displaying the greatest likelihood of achieving a pathological complete response.

Using pathological evaluation of lymphocytes and tandem LCM followed by gene expression analysis of adjacent tumor and stromal cells, we demonstrated that the previously described IM and MSL subtypes are composed of tumors with low cellularity. Therefore, we have refined TNBC from six to four (TNBCtype-4) transcriptional subtypes, using IM and MSL correlations as subtype descriptors of cellular heterogeneity. Refined TNBCtype-4 subtypes show consistencies with histological differences observed by pathologists, such as lobular breast cancers belonging to the LAR subtype and metaplastic breast cancers having a BL2 or M subtype. Consistent with differing biologies and histologies, are differences in disease progression and metastatic spread. Paradoxically, BL1 tumors are higher grade but lower stage than LAR. There is an increased frequency of regional lymph node involvement in LAR TNBC and differential metastatic spread to the lung and bone for M and LAR tumors, respectively. This tissue tropism likely reflects unique tumor biology and suggests the need for different and more comprehensive approaches for monitoring metastatic disease in newly diagnosed M and LAR patients, with more personalized imaging approaches.

Approximately 20% of TNBCs classify as immunomodulatory and are highly enriched in immune cell makers and signaling. Pathological evaluation of lymphocytes from H&E sections provide significant evidence that infiltrating lymphocytes within tumors drives the overall gene expression of the IM subtype. The presence of tumor-associated lymphocytes in a TNBC generated a gene expression profile that had increased expression of immune checkpoint regulators such as PD1, PD-L1 and CTLA4, strongly correlated with the IM gene signature centroid and was associated with increased relapse-free survival for the patient. These data are of interest, given the promising phase I results with anti-PDL1 inhibitors, in which 18.5% of 27 TNBC patients responded to pembrolizumab[31].

Of note, recent studies have shown that the presence of TILs are associated with better response to adjuvant chemotherapy[32] and neoadjuvant chemotherapy[23]**.** This association with neoadjuvant response appears to be more pronounced with platinum based agents, as patients enrolled in the doxorubicin with carboplatin arm of the GeparSixto (GBG 66) trial had a greater odds of achieving pCR than those on the doxorubicin arm alone[23].

The IM subtype descriptor has the potential to be a semi-quantitative biomarker for immune-reactive TNBC tumors and consideration should be given to investigating if it can identify patients that may benefit from immune checkpoint inhibitors. Interestingly, select tumors representative of all the subtypes, except M, had some degree of correlation to the IM centroid and presence of immune cells. In fact, there was a strong negative correlation between the IM and M subtypes, suggesting M tumors create a microenvironment that is immune-suppressive.

Certain TNBC patients clearly benefit from chemotherapy that includes a combination of anthracylines, alkylating agents and microtubule inhibitors in the neoadjuvant, adjuvant and metastatic settings. However, historically this benefit is restricted to a subset of patients, with approximately 22–30% achieving pCR in the neoadjuvant setting that correlates with overall and event free survival[12]. Currently, no examples of validated predictive biomarkers for individual chemotherapeutics have been described outside of platinum agents for BRCA1/2-mutated TNBC[6,33,34].

Since TNBC subtypes have previously been shown to be independently associated with pCR, we re-evaluated response to neoadjuvant chemotherapy in TNBC patients from five neoadjuvant chemotherapy datasets stratified into the refined TNBCtype-4 molecular subtypes. Initial evaluation of the GSE25066 dataset was consistent with the previous publication, in which BL1 had the highest pCR and BL2 and LAR the lowest[13]. Similar evaluation of tumors stratified by PAM50 showed basal tumors having a better response than non-basal. The decreased response of BL2 tumors to neoadjuvant chemotherapy was consistent with decreased distant relapse-free survival for those patients. In contrast, the LAR subtypes had better survival despite a decreased response to neoadjuvant chemotherapy. The decreased response of AR-positive TNBC tumors to neoadjuvant chemotherapy has recently been validated with the report of significantly lower pCR and increased disease recurrence in AR-positive TNBC patients[35]. The discrepancy between response and survival in the LAR subtype can potentially be explained by the decreased proliferation and well-differentiated luminal state of this subtype.

Combined analysis of over 300 TNBC patients receiving neoadjuvant chemotherapy showed that BL2 patients were significantly less likely to achieve a pCR than TNBC as a whole. These data are also supported by decreased relapse-free survival in the GSE25066 cohort, with a less than a 3 y median survival compared to a 7 y for unselected TNBC, and highlight the unmet medical need to identify alternative therapeutic strategies for this patient population. In contrast, BL1 patients were nearly 50% more likely to achieve a pCR compared to unselected TNBC. Even though BL1 tumors are more likely to be of higher grade, they are more responsive in general to genotoxic chemotherapies. The latter is likely due, in part, to aberrant DNA signaling and repair functions in the BL1 subtype tumors. Majority of the samples were classified as BL1 (36%), however after neoadjuvant chemotherapy, one would anticipate a greater enrichment of chemo-insensitive subtypes, such as BL2 and LAR, in patients with residual disease. Stratification into the BL1 subtype may identify a patient population more responsive to chemotherapy and those patients for whom chemotherapeutic treatments are most appropriate.

## Conclusions

Our analyses and resulting data refine TNBC into four molecular subtypes and provide further evidence that patients with tumors subtyped as BL1 will receive greater benefit from standard neoadjuvant chemotherapy such as ACT than patients with other TNBC subtypes such as BL2 and LAR. Subtyping of TNBC tumors should provide significant value for future clinical decision-making and the alignment of TNBC patients with traditional chemotherapy versus targeted and immune-based therapies that are currently under clinical investigation.

## Supporting Information

S1 Fig The M and IM subtype correlations are inversely correlated. **(A)** Scatter plots show pairwise correlation values for 587 TNBC tumors and the six TNBC molecular subtypes. **(B)** Density plots show the frequency of subtype correlation strength across the six TNBC centroids by increasing correlation strength. IM = immunodulatory, BL1 = basal-like 1, BL2 = basal-like 2, MSL = mesenchymal stem-like, M = mesenchymal and LAR = luminal androgen receptor.
(TIF)

S2 Fig Differential gene expression in adjacent tumor epithelium and stroma from 10 TNBC patients. Principal component analysis (PCA) plots show **(A)** individual pairs of tumor (T) and stroma (S) isolated from the same patient (color) or **(B)** the separation of gene expression between tumor and stromal samples. The first two components (Dim 1 and Dim 2) describe 93.6% of the variation between samples.
(TIF)

S3 Fig Correlation to the IM subtype is significantly associated with longer overall survival. Plots show COX proportional hazard modeling IM subtype correlation as a function of time [Beta(t)] with 95% confidence interval (dotted lines) using **(A)** relapse-free survival (RFS) and **(B)** overall survival (OS). Indicated p-values were determined by likelihood ratio tests.
(TIF)

S4 Fig Identification of TNBC patients by mixed-Gaussian distribution. Beeswarm plots show expression (mRNA) for ESR, PGR and ERBB2 for individual tumors from **(A)** GSE25066 **(B)** GSE41998 **(C)** GSE22226 (GPL1708), **(D)** GSE22226 (GPL4133) and **(E)** GSE22358. Data points are colored by pathological calls for ER, PR and HER as determined by immunohistochemistry or fluorescence in situ hybridization to be positive (red) or negative (black). Dotted line indicates bimodal mixed Guassian distribution cutoff for positivity.
(TIF)

S5 Fig Response to neoadjuvant chemotherapy in TNBC stratified by subtypes. Barplots show pCR rates achieved in four clinical trials in which patients were stratified by clinical subtype into non-TNBC and TNBC (**A, D, G, J, M**), into basal and non-basal by PAM50 molecular subtype (**B, E, H, K** and **N**) or into BL1, BL2, M and LAR by refined TNBCtype-4 (**C, F, I, L** and **O**). Dotted horizontal line indicates pCR for the overall cohort. A-T, sequential anthracycline and taxane; P-FEC, paclitaxel followed by a combination of 5-fluorouracil, epirubicin and cyclophosphamide; AC-T/IXA, anthracycline and cyclophosphamide followed by either taxane or ixabepilone.
(TIF)

S1 Table Comparison of pathology scoring of TCGA H&E stained for TILs relative to TNBC molecular subtypes and centroid correlation. (XLSX)

S2 Table Differentially expressed genes in tumor epithelium compared to adjacent tumor stroma. (XLSX)

S3 Table Clinical annotations for TCGA and TNBC587 samples. (XLSX)

S4 Table Distribution of TNBCtype, TNBCtype-4 and PAM50 subtypes in 587 TNBC microarray profiles and 180 TNBC tumors from TCGA. (XLSX)

S5 Table Evaluation of metastasis site by TNBC subtypes. (XLSX)

S6 Table Atypical histological subtype distribution among TNBC molecular subtypes. (XLSX)

S7 Table Clinical information for neoadjuvant chemotherapy datasets. (XLSX)

## Abbreviations

A: Anthracycline
BL1: basal-like1
BL2: basal-like 2
C: cyclophosphamide
DRFS: distant relapse-free survival
ER: estrogen receptor
HRD: homologous recombination deficiency
HER2: human epidermal growth factor receptor 2
IM: immunomodulatory
LCM: laser-capture microdissection
LAR: luminal AR
M: mesenchymal
MSL: mesenchymal stem-like
OR: odds ratio
pCR: pathological complete response
PR: progesterone receptor
RD: residual disease
T: taxane
TCGA: The Cancer Genome Atlas
TNBC: triple negative breast cancer
TILs: tumor infiltrating lymphocytes
