# Supplementary figures and images for: Refinement of Triple-Negative Breast Cancer Molecular Subtypes: Implications for Neoadjuvant Chemotherapy Selection
Source: PLoS One. 2016 Jun 16;11(6):e0157368. doi: 10.1371/journal.pone.0157368 (PMC4911051; doi:10.1371/journal.pone.0157368)

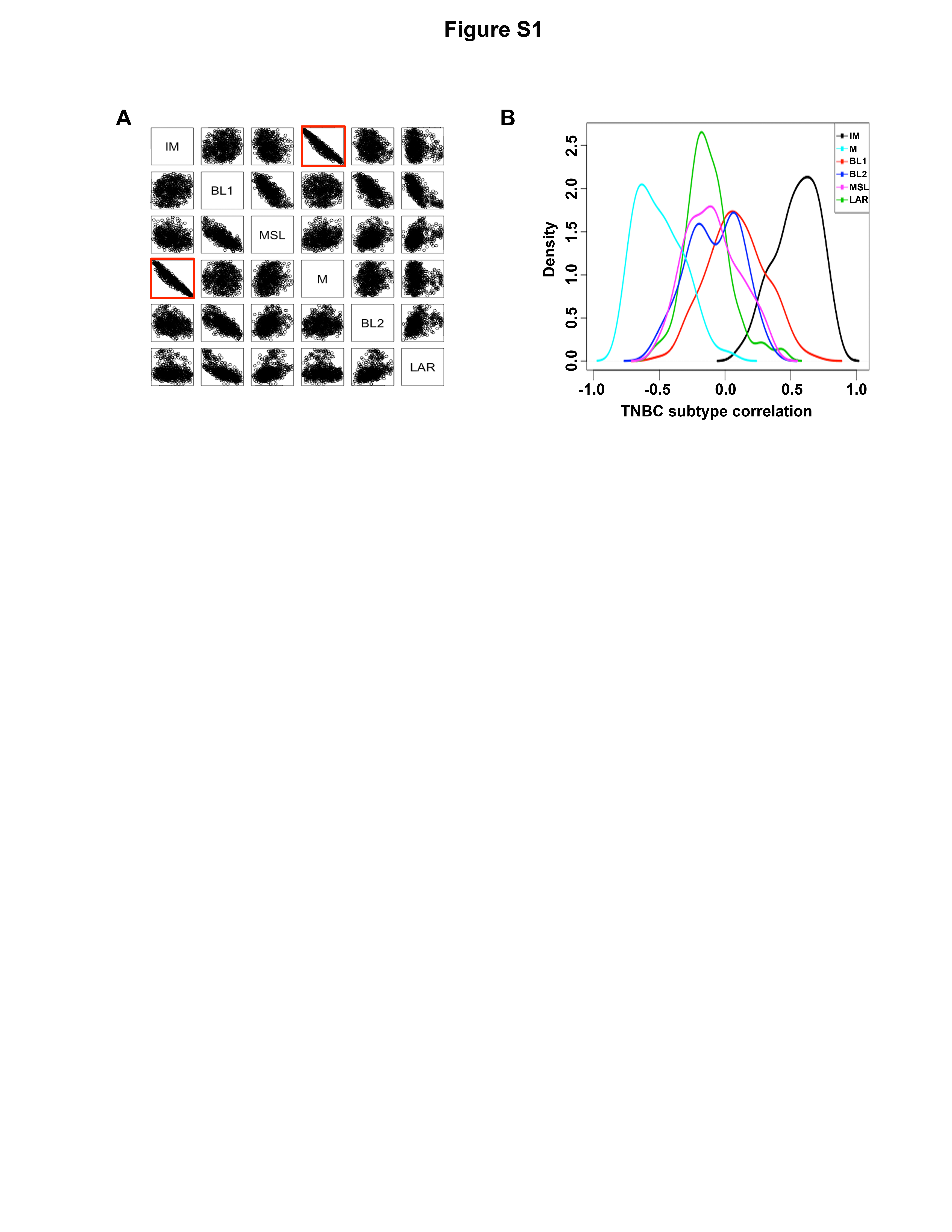

Supplement: S1 Fig — (A) Scatter plots show pairwise correlation values for 587 TNBC tumors and the six TNBC molecular subtypes. (B) Density plots show the frequency of subtype correlation strength across the six TNBC centroids by increasing correlation strength. IM = immunodulatory, BL1 = basal-like 1, BL2 = basal-like 2, MSL = mesenchymal stem-like, M = mesenchymal and LAR = luminal androgen receptor. (TIF) [file pone.0157368.s001.tif]

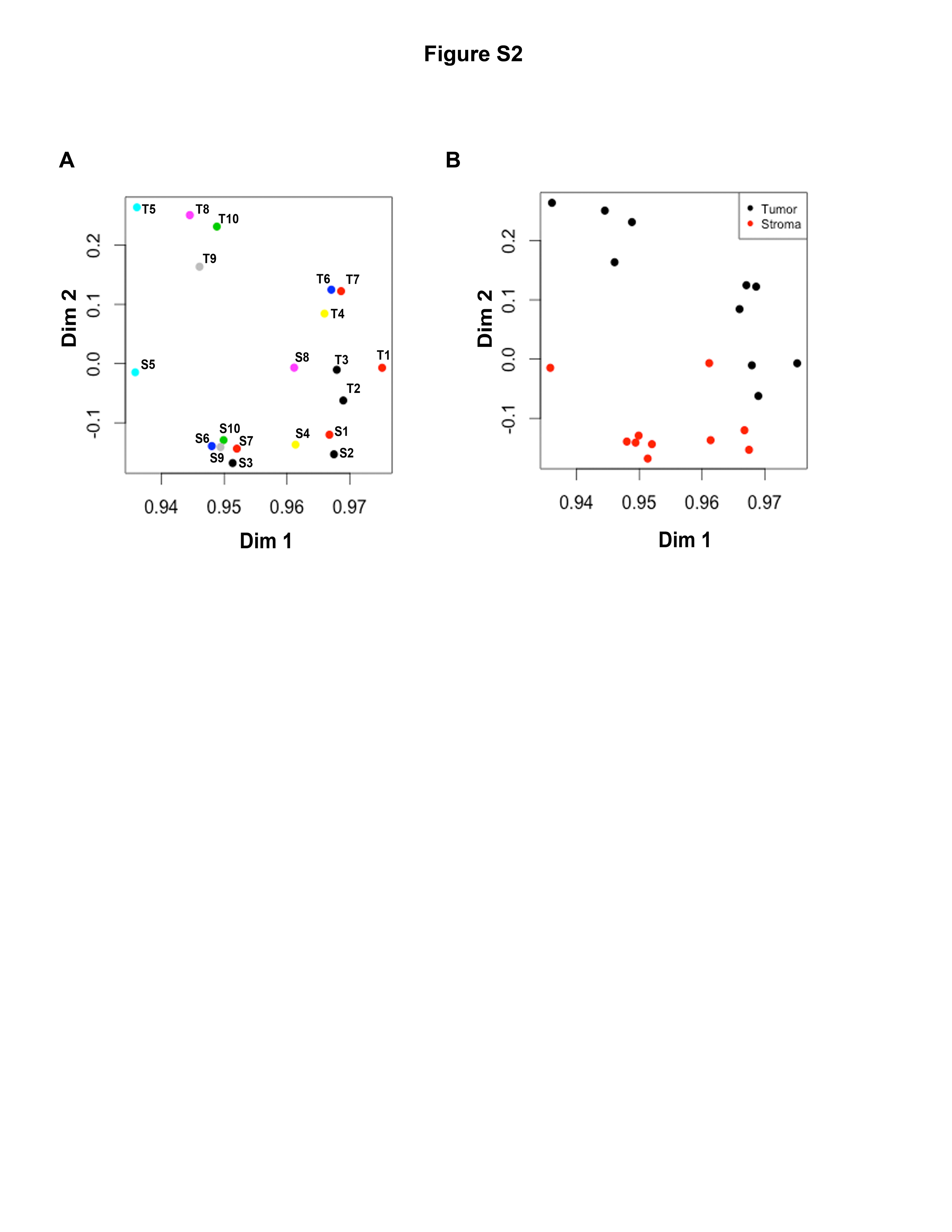

Supplement: S2 Fig — Principal component analysis (PCA) plots show (A) individual pairs of tumor (T) and stroma (S) isolated from the same patient (color) or (B) the separation of gene expression between tumor and stromal samples. The first two components (Dim 1 and Dim 2) describe 93.6% of the variation between samples. (TIF) [file pone.0157368.s002.tif]

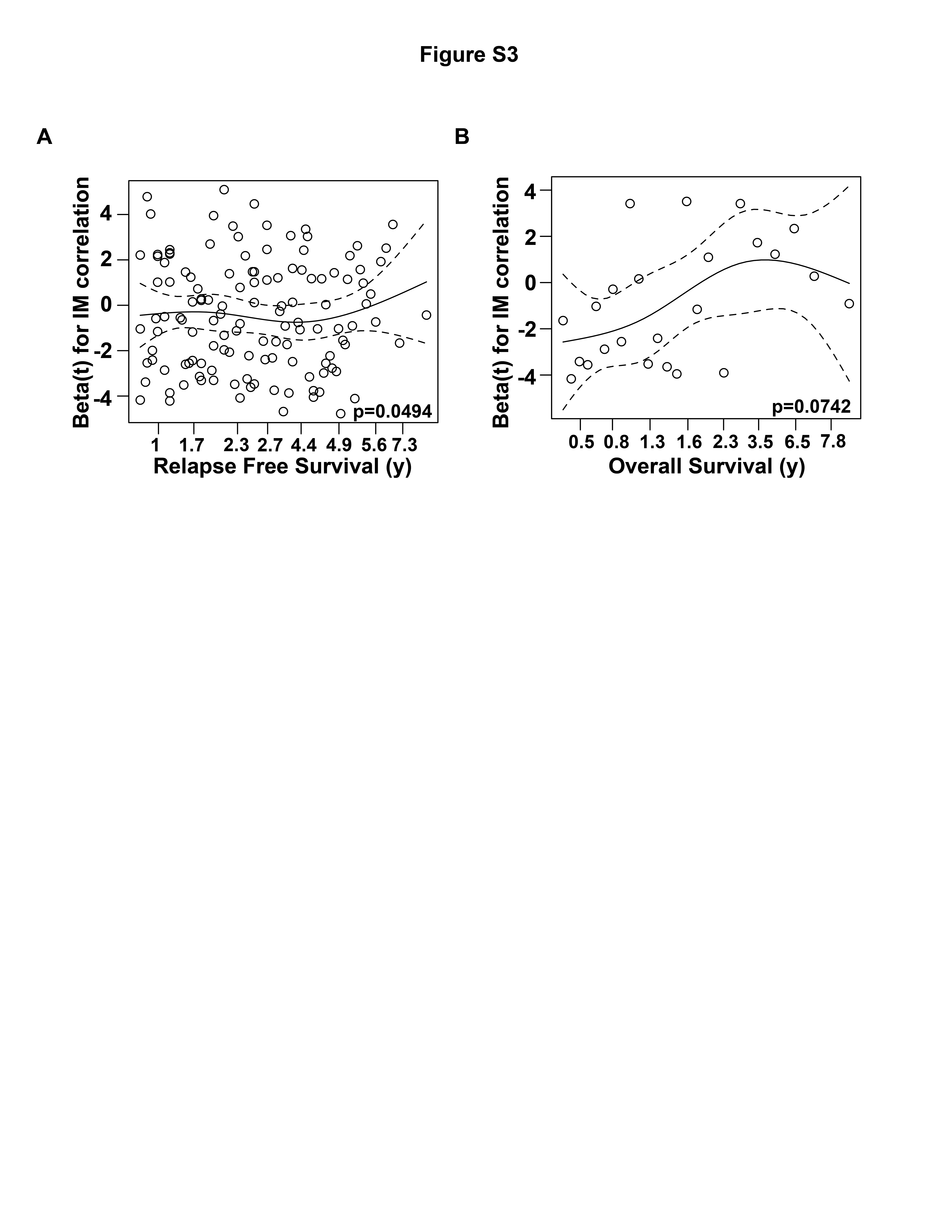

Supplement: S3 Fig — Plots show COX proportional hazard modeling IM subtype correlation as a function of time [Beta(t)] with 95% confidence interval (dotted lines) using (A) relapse-free survival (RFS) and (B) overall survival (OS). Indicated p-values were determined by likelihood ratio tests. (TIF) [file pone.0157368.s003.tif]

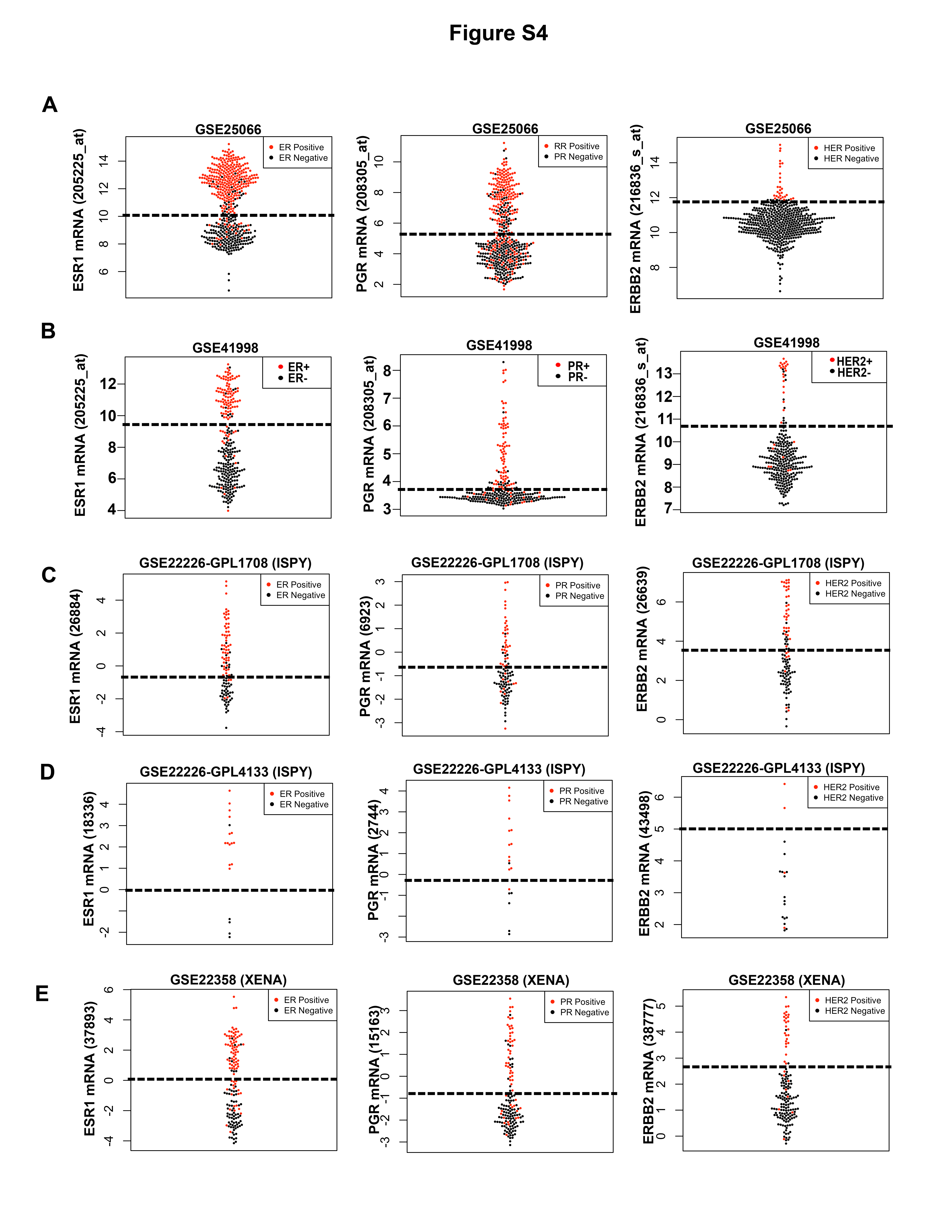

Supplement: S4 Fig — Beeswarm plots show expression (mRNA) for ESR, PGR and ERBB2 for individual tumors from (A) GSE25066 (B) GSE41998 (C) GSE22226 (GPL1708), (D) GSE22226 (GPL4133) and (E) GSE22358. Data points are colored by pathological calls for ER, PR and HER as determined by immunohistochemistry or fluorescence in situ hybridization to be positive (red) or negative (black). Dotted line indicates bimodal mixed Guassian distribution cutoff for positivity. (TIF) [file pone.0157368.s004.tif]

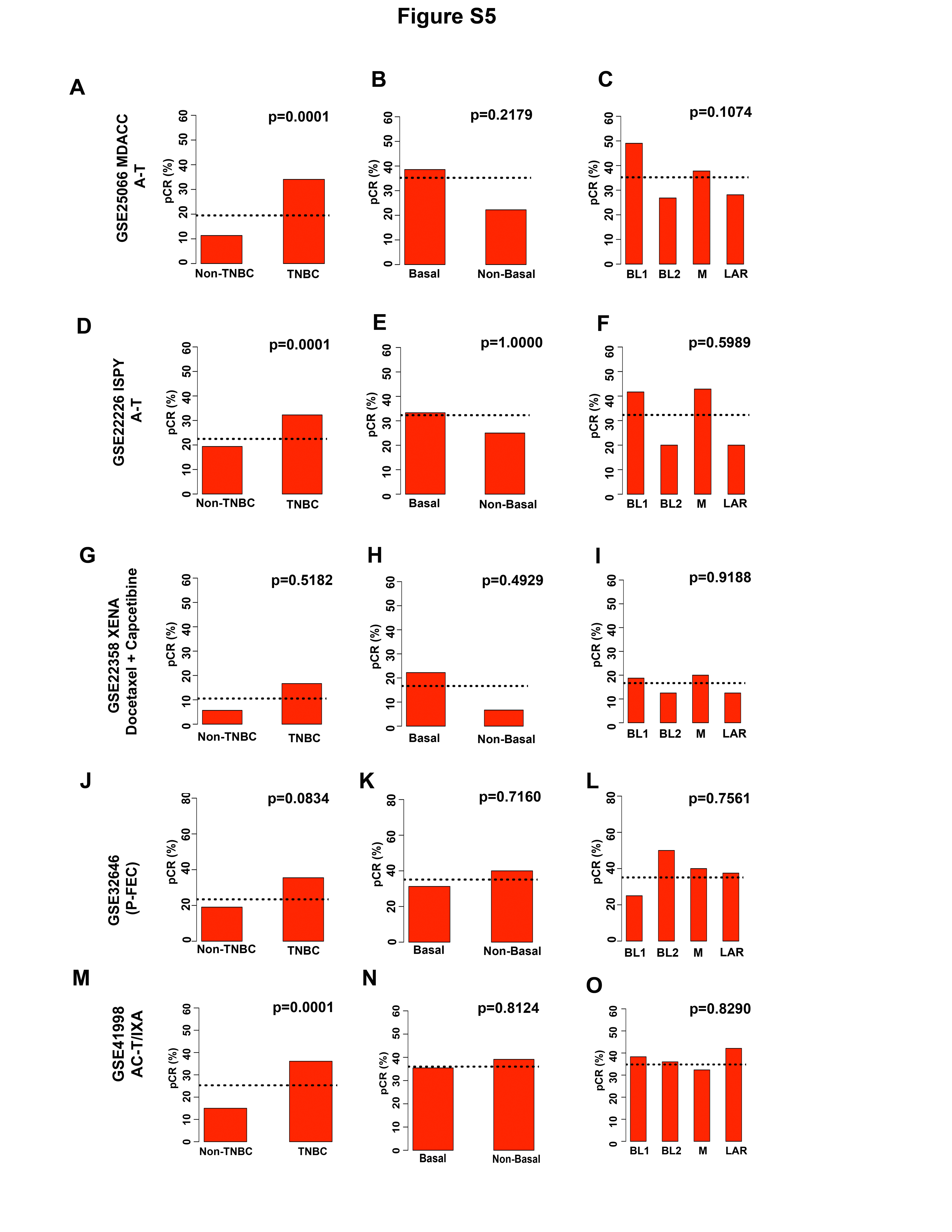

Supplement: S5 Fig — Barplots show pCR rates achieved in four clinical trials in which patients were stratified by clinical subtype into non-TNBC and TNBC (A, D, G, J, M), into basal and non-basal by PAM50 molecular subtype (B, E, H, K and N) or into BL1, BL2, M and LAR by refined TNBCtype-4 (C, F, I, L and O). Dotted horizontal line indicates pCR for the overall cohort. A-T, sequential anthracycline and taxane; P-FEC, paclitaxel followed by a combination of 5-fluorouracil, epirubicin and cyclophosphamide; AC-T/IXA, anthracycline and cyclophosphamide followed by either taxane or ixabepilone. (TIF) [file pone.0157368.s005.tif]
